# Circadian-tunable Perovskite Quantum Dot-based Down-Converted Multi-Package White LED with a Color Fidelity Index over 90

**DOI:** 10.1038/s41598-017-03063-7

**Published:** 2017-06-05

**Authors:** Hee Chang Yoon, Ji Hye Oh, Soyoung Lee, Jae Byung Park, Young Rag Do

**Affiliations:** 1 Department of Chemistry, Kookmin University, Seoul, 136-702 Republic of Korea; 2 Department of Display and Semiconductor Physics, Korea University, Sejong, 30019 Republic of Korea

## Abstract

New metrics of the color and circadian performances of down-converted white light-emitting diodes (DC-WLEDs) are rapidly becoming popular in smart lighting systems. This is due to the increased desire for accurate analytical methods to measure the effects of newly developed quantum dot (QD)-based lighting on the vision, color, and circadian sensors of retina cells in the human eye. In this regard, a two-measure system known as technical memorandum TM-30-2015 (Illuminating Engineering Society of North America), encompassing the color fidelity index (CFI, *R*_*f*_) and the color gamut index (CGI, *R*_*g*_), has been developed as a new metrics of color to replace the currently utilized color rendering index (CRI, *R*_*a*_). In addition, the tunability of the circadian efficacy of radiation (CER) is now more important due to its effect on the control of melatonin suppression/secretion, resetting of the central/local clocks of individuals given their daily cycles, and benefits to human health. In this paper, we developed and analyzed six-colored perovskite (Pe; cyan, green, yellowish green, amber, orange, and red colors) QDs-based multi-package WLED, and optimized the SPDs of tunable PeQD-based multi-package WLEDs in terms of promising human-centric lighting device, given its optimized visual energy, color qualities and health-promoting effects.

## Introduction

**Figure 1:**
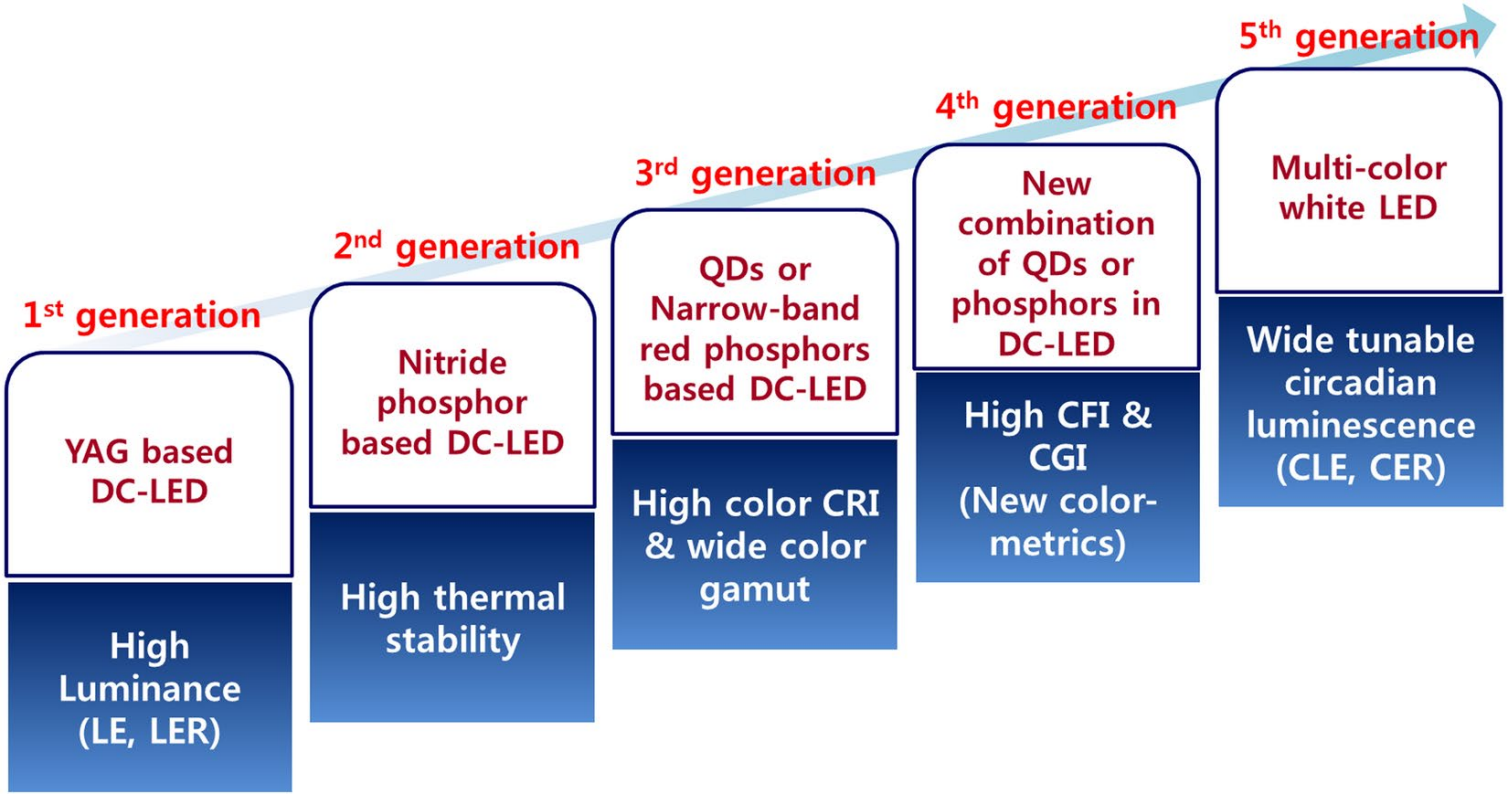
Schematic diagram of the development approaches for the first through fifth generations for general WLED lighting devices. [Luminous efficacy (LE), luminous efficacy of radiation (LER), color rendering index (CRI), color fidelity index (CFI), color gamut index (CGI), circadian luminous efficacy (CLE), and circadian luminous efficacy of radiation (CER)].

Solid-state lighting types are increasing in the general lighting market due to their energy savings, extended longevity and eco-friendly characteristics^1, 2^. Two major performance metrics, the color quality (color rendering index, CRI or *R*_*a*_) and the visual energy efficiency (luminous efficacy of radiation, LER), are the critical criteria to be considered when developing luminescent materials for down-converted white-light-emitting diodes (DC-WLEDs)^3^. A high LER and a high *R*_*a*_ serve as target figures of merit in the early stages of the development of WLEDs between the first and third generation of general WLED lightings (see Fig. 1). In this regard, various DC quantum dots (QDs) have recently been developed with maximized CRI and LER values as well as extended longevity to replace the currently commercialized rare-earth-based inorganic phosphors for DC-WLEDs^4–6^. At present, the CRI is well known as a color metric, though it is limited when used to reproduce good saturated colors of illuminated objects under white conditions. Fairly recently, the Illuminating Engineering Society of North America developed and adopted a two-measure system with a color fidelity index (CFI, *R*_*f*_) and a color gamut index (CGI, *R*_*g*_) as technical memorandum TM-30-2015 in the fourth generation of the development of WLEDs for accurate evaluations of the color rendition and the color performances of DC-WLED lighting sources (see Fig. 1)^7, 8^. In this regard, we recently reported how various narrow-band red phosphors affect the new color metrics of warm white light from tri-color DC-WLEDs^9^. As an unexpected result of comparing the currently used CRI color metric and the newly developed color metric, it was found to be very difficult for any of the tri-color DC-WLEDs with any type of narrow- or broad-band red phosphors to reach an *R*_*f*_ score exceeding 90 owing to the overestimation of *R*_*a*_ scores over 90. Put simply, any spectral power distributions (SPDs) of warm white LEDs with *R*_*a*_ scores exceeding 90 cannot be correlated with an *R*_*f*_ score over 90. Hence, it is necessary to reconsider the development strategies used when choosing DC materials such as QDs or inorganic phosphors as well as the related color-mixing strategies, such as the number of colors and the broadness or narrowness of the photoluminescence (PL) bandwidth and optimum peak wavelength of individual color spectra when formulating the high color qualities of all white colors in the range of cool white to warm WLEDs.

In addition to both a high color quality level for color perception/reproduction and high visual efficiency to reduce energy use, another important figure of merit for smart WLEDs is the tunable capability of the circadian energy efficiency (circadian efficacy of radiation, CER) for entraining the central and local clocks of the human body with the circadian rhythm^3^. The tunability of the circadian effect has obtained greater prominence in recent years for controlling melatonin suppression/secretion, resetting the central/local clocks of individuals, and improving human health by matching the spectrum of QD-containing WLEDs with daily variations of sunlight under the natural circadian rhythm^10–12^. In the long run, circadian-controlled lightings will become a mainstream type of human-centric lighting and a main performance target in the fifth generation of developing DC-QD materials for DC-WLEDs, as shown in Fig. 1.

Two newly proposed figures of merit, the two-measure system of colors (*R*_*f*_ and *R*_*g*_) and the circadian tunability of the CER, should also be analyzed and optimized to develop appropriate DC-QD materials for highly efficient, widely color-reproducible and healthful DC-WLED lightings. As shown in Fig. 1, high luminance (first generation), high thermal stability (second generation) and high CRI (third generation) were sequentially developed by DC materials and LED-related scientists and engineers to produce illumination-grade LED lightings before the two-measure system of color metrics and the circadian-tunable system to control circadian luminance levels were considered. Therefore, a high quantum yield (QY), pure color coordinates, and tunable PL peaks must initially be considered as prerequisites in the development of DC-QD materials for energy-saving, human-centric, and sun-like color-reproducible WLED lamps. These prerequisites are described in detail below; the peak wavelength of QDs should be tunable from cyan to deep red in the visible wavelength range to realize multi-package white-by-blue LEDs, and the bandwidth of the PL spectrum and the number of color channels should be optimized for efficient, color-reproducible, healthful, and tunable WLEDs.

**Figure 2:**
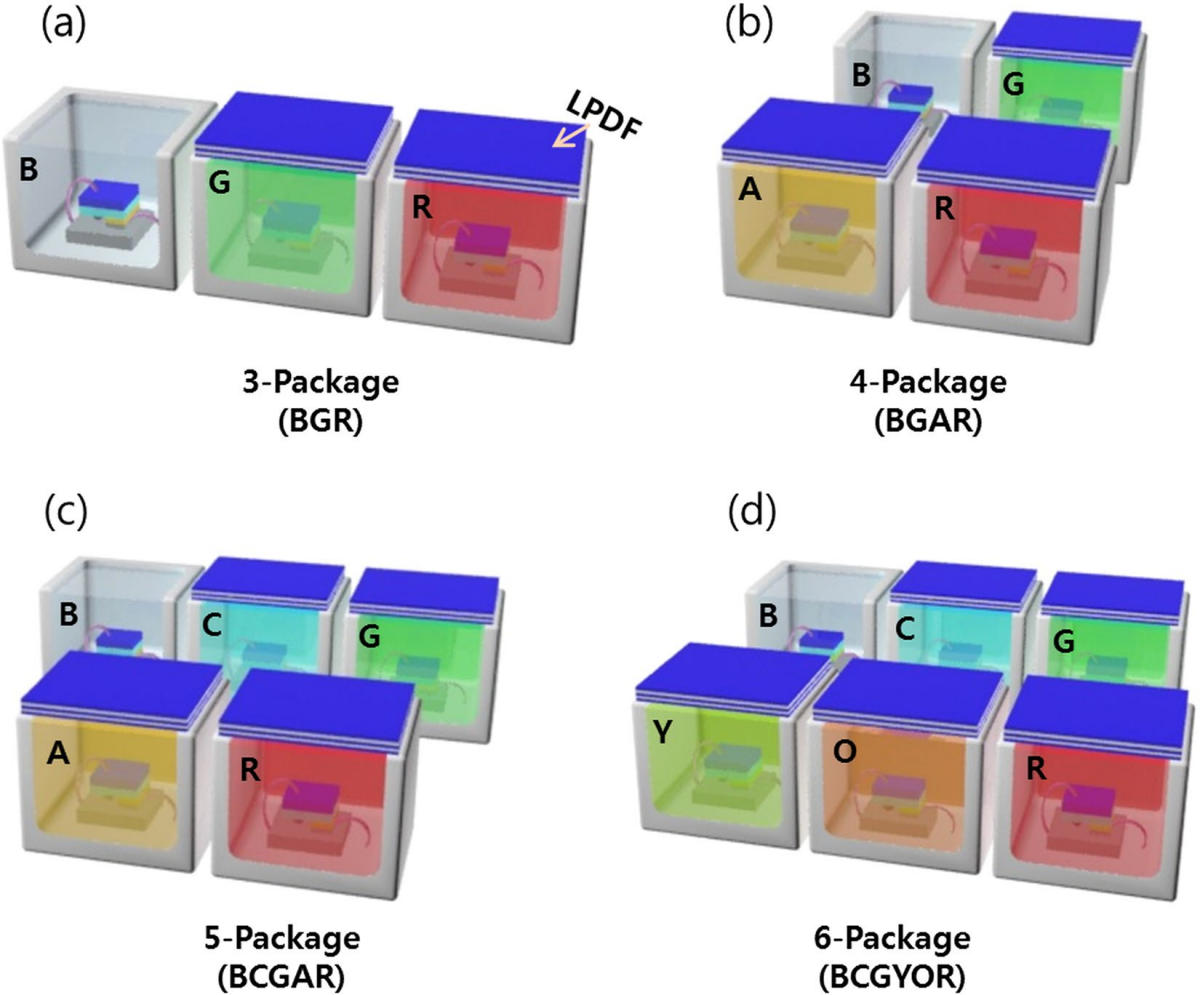
Schematic diagram of four types of multi-package white LEDs incorporating colored PeQDs.

Recently developed perovskite (Pe) QDs as well as Cd-containing II-VI QDs are excellent candidates for tunable multi-package WLEDs^13–17^. Instead of using environmentally harmful, recently commercialized Cd-containing II-VI QDs, the PeQD family is becoming more popular as a less environmentally harmful and excellent candidate for maximizing the circadian tunable range and two-measure system of color quality levels. Furthermore, it is necessary to analyze how an efficient LER value, a tunable CER range, a high *R*_*f*_ score exceeding 90, and an *R*_*g*_ score close to 100 can be obtained from multi-package WLEDs incorporating tunable PeQDs while optimizing the number of mixed monochromatic colors from three to six DC-LEDs (one blue-chip LED plus two or two more fully converted QD-LEDs). In this study, we synthesized six different colors (cyan (C), green (G), yellowish green (Y), amber (A), orange (O) and red (R)) of CsPb(Br_1−x_, X_x_)_3_ (X = Cl, Br) PeQDs using a hot-injection method developed in our previous study^17^ and fabricated five PeQD-based CGYOR-emitting monochromatic DC-LEDs and a multi-package WLED based on them with a blue (B)-chip LED. We analyzed and optimized the SPDs of tunable multi-package WLEDs in terms of energy savings, color reproducibility and health benefits. Here, multi-channel LED clusters were prepared from tri- to six-color multi-package LEDs (BGR, BGAR, BCGAR, and BCGYOR), as shown in the schematic diagram of the multi-package WLEDs in Fig. 2. In detail, we also analyzed the non-visual circadian, color-related, and visual figures of merit of the multi-package WLEDs, specifically the circadian efficacy of radiation (CER), the circadian action factor (CAF), the color fidelity index (CFI, *R*_*f*_), the color gamut index (CGI, *R*_*g*_), the color icon graph, and the luminous efficacy of radiation (LER) within a correlated color temperature (CCT) tunable range of 2000 K to 10000 K to mimic the sunlight spectrum and control the circadian luminance according to the 24-hr rhythmic change of the natural light environment and to determine the possibility of exceeding a CFI value of 90 in the white color range between warm and cool white.

## Results and Discussion

**Figure 3:**
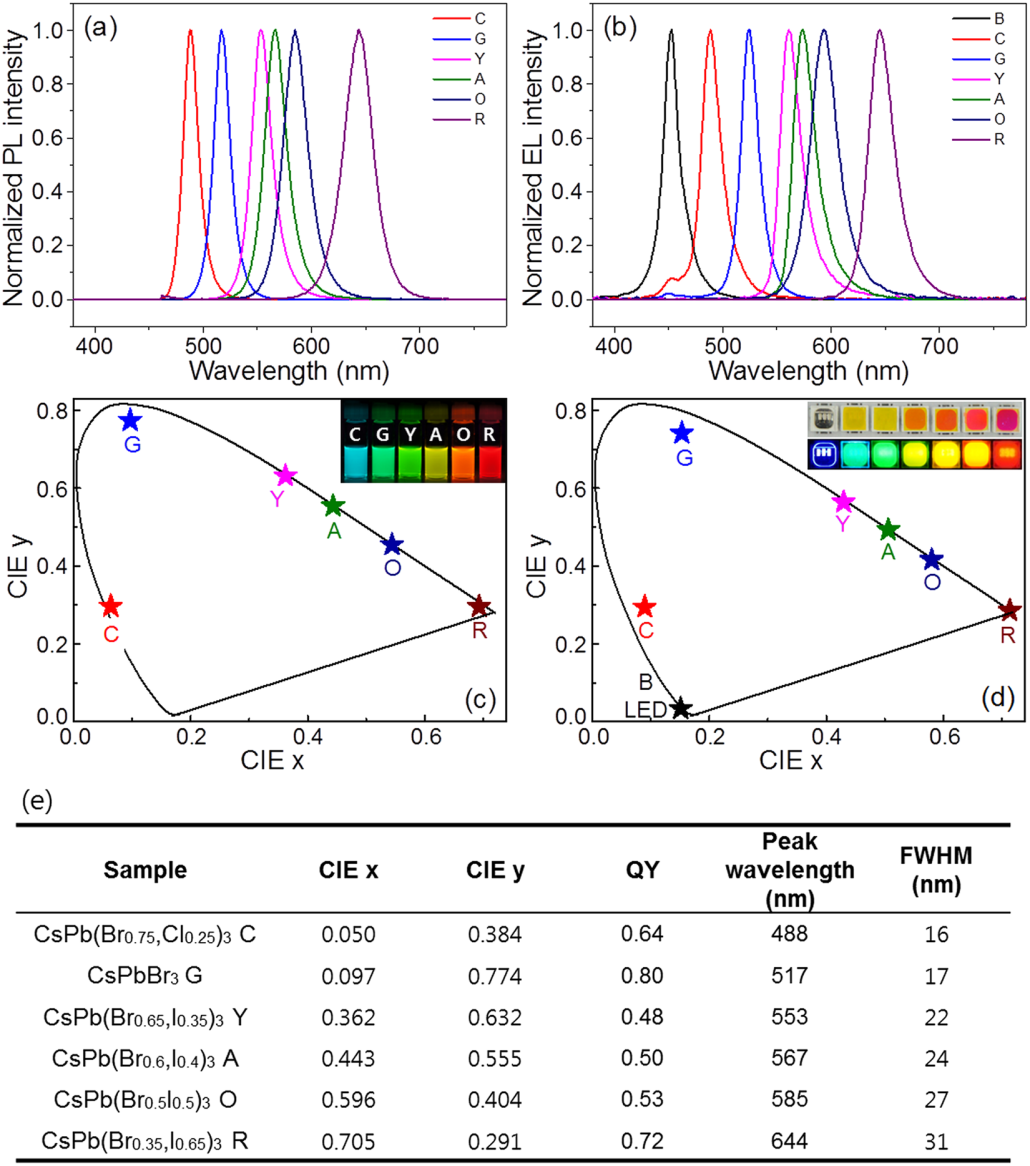
(**a**) PL spectra of CGYAOR-emitting CsPb(Br_1−x_, X_x_)_3_ PeQDs and (**b**) EL spectra of PeQD-based CGYAOR-emitting monochromatic DC-LEDs and InGaN B-chip LEDs. CIE color coordinates of (**c**) PeQDs and (**d**) PeQD-based monochromatic DC-LEDs. The insets of (**c**) and (**d**) show emission photographs of the six-color colloidal PeQDs and LPDF-capped PeQD package DC-LEDs. (**e**) Optical properties of colloidal CsPb(Br_0.75_, Cl_0.25_)_3_ cyan, CsPbBr_3_ green, CsPb(Br_0.65_, I_0.35_)_3_ yellowish green, CsPb(Br_0.6_, I_0.4_)_3_ yellow, CsPb(Br_0.5_, I_0.5_)_3_ orange, and CsPb(Br_0.35_, I_0.65_)_3_ red PeQDs.

Tunable CsPb(Br_1−x_, X_x_)_3_ PeQDs were synthesized by controlling the halide anion contents and reaction temperatures to obtain six different colors of CGYAOR-emitting CsPb(Br_1−x_, X_x_)_3_ PeQDs. The detailed optical and crystal results of the CGYAOR-emitting PeQDs are described in Figure S1 in the Supporting Information. Based on the PL analysis, six CsPb(Br_0.75_, Cl_0.25_)_3_, CsPbBr_3_, CsPb(Br_0.65_, I_0.35_)_3_, CsPb(Br_0.6_, I_0.4_)_3_, CsPb(Br_0.5_, I_0.5_)_3_, and CsPb(Br_0.35_, I_0.65_)_3_ PeQDs were synthesized as CGYAOR-emitting PeQDs for application into CGYAOR-emitting monochromatic DC-LEDs. Figure 3a,b show the PL spectra of the CGYAOR-emitting PeQDs and the DC-electroluminescent (EL) spectra of their monochromatic CGYAOR-emitting DC-LEDs along with a B chip LED. As previously reported^12–14^, the color tunability characteristics of PeQDs were obtained from the blue-shift of the PL emission energies of the PeQDs by increasing the concentration of the smaller anion (I → Br → Cl) constituent. Here, the PL colors of the PeQDs can be blue-shifted by widening the bandgap through the formation of an alloy with smaller anions, decreasing both the unit cell and the covalency of the PeQDs. As we reported previously^17–19^, six selected colors of fully down-converted LEDs were fabricated by capping a LPDF-capped LED package filled with PeQD/NOA 63 paste. As shown in Fig. 3b, the emission wavelengths of the six CGYAOR-emitting DC-LEDs reached 489, 525, 561, 574, 593, and 645 nm with (Full-width at half-maximum) FWHM values of 21, 19, 23, 24, 30, and 28 nm, respectively. The DC-EL emission spectra and FWHM values of the CGYAOR-emitting monochromatic DC-LEDs are well matched with those of the corresponding PeQDs, as previously reported^14, 16, 17^, although the peak wavelengths are slightly red-shifted. This slight red shift from QDs to QD-based DC-LEDs is assumed to have resulted from PeQD agglomeration and energy exchange between neighboring PeQDs in the PeQD/NOA 63 binder package of the LPDF-capped LEDs^18–21^. (See Figure S2) The color-emitting images of the five DC-LEDs also confirm that pure color of ELs can be realized using the color-by-blue concept with help of the LPDF and a series of narrow-band CsPb(Br_1−x_, X_x_)_3_ PeQDs. Figure 3c,d show the 1931 CIE color coordinates of the six selected PeQDs and the B semiconductor-type LED along with the six fully down-converted, LPDF-capped PeQDs-based DC-LEDs. The color gamut area of the blue LED and the five CGYOR hexagons of the monochromatic LEDs and PeQDs cover ~145% and ~162% of the NTSC standard, respectively. The combination of the five CGYOR monochromatic LEDs and a B-chip LED can produce the largest ever color reproduction area levels with artificial lighting and can control the finest ever white color and mixed colors through the tuning of multi-package WLEDs.

**Figure 4:**
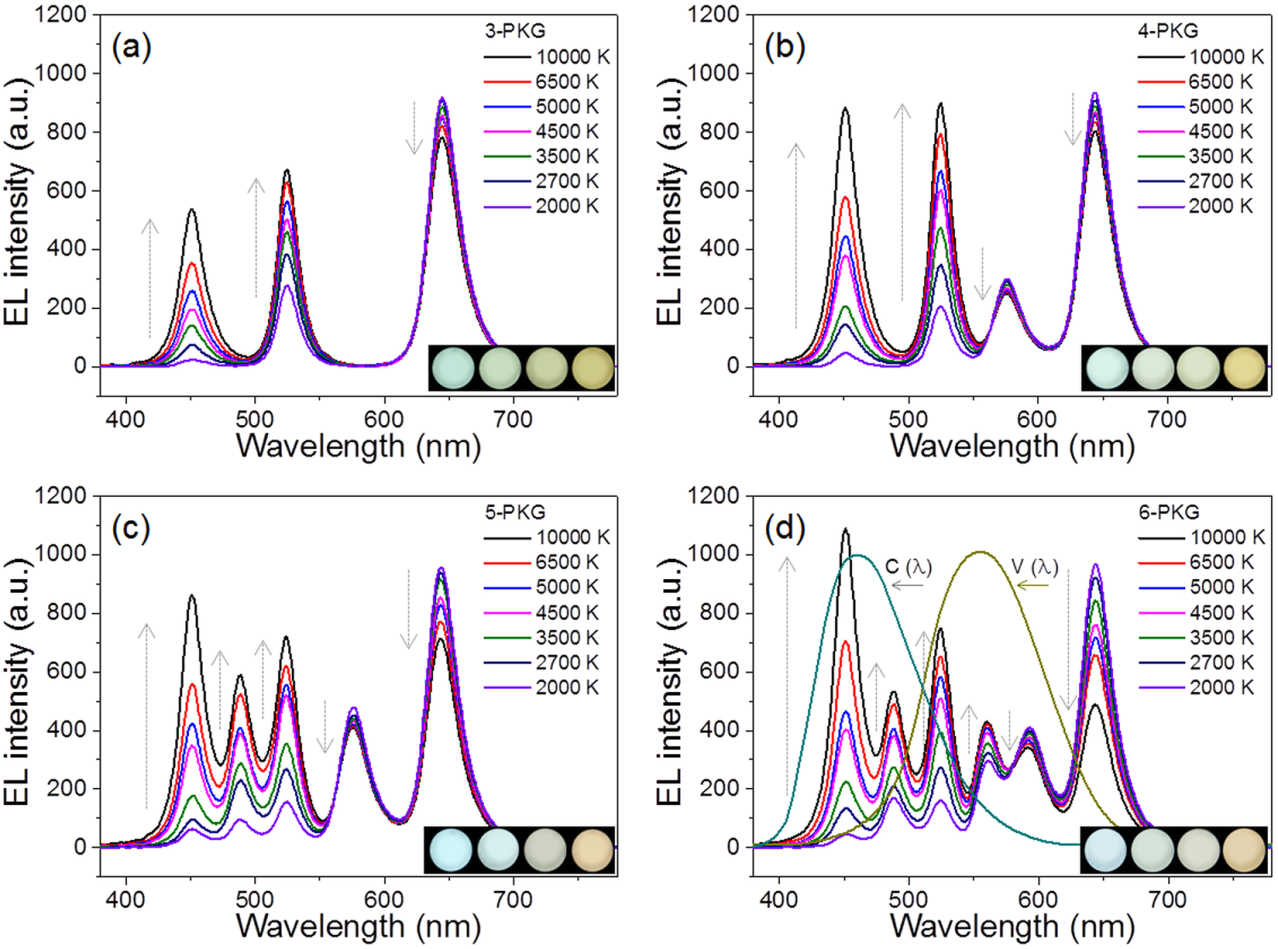
EL spectra of (**a**) three-, (**b**) four-, (**c**) five-, and (**d**) six-package white LEDs in a white CCT ranging between 10000 K to 2000 K. The gray arrows indicate an increase or a decrease of the EL intensity level with an increase in the CCT. The cyan and dark yellow spectra refer to the circadian spectral function [C(λ)] and the photopic spectral function [V(λ)], respectively. Insets show actual emission images of each multi-package white light from a black-body sphere with an increase in CCT from right (3500 K) to left (6500 K).

As summarized in Fig. 3e, the CsPb(Br_0.75_, Cl_0.25_)_3_, CsPbBr_3_, CsPb(Br_0.65_, I_0.35_)_3_, CsPb(Br_0.6_, I_0.4_)_3_, CsPb(Br_0.5_, I_0.5_)_3_, and CsPb(Br_0.35_, I_0.65_)_3_ PeQDs provide PLQYs of 0.64, 0.80, 0.48, 0.50, 053, and 0.72, respectively, and their corresponding CGYAOR monochromatic DC-LEDs provide luminous efficacy (LE) values of 49, 169, 86, 100, 86, and 25 lm/W at an applied current of 20 mA. These PLQY and LE values of PeQD and DC-LEDs are in good agreement with those in previous publications^17, 20^. Here, three-, four-, five-, and six-package DC-WLEDs were prepared and characterized while combining parts of six differently colored DC-LEDs and a B chip LED to compare the new metrics of the color properties and the circadian tunability of the multi-package WLEDs. Four types (BGR, BGAR, BCGAR, and BCGYOR) of multi-package WLEDs can realize a set of seven correlated color temperatures (CCTs), as specified in the American National Standards Institute (ANSI) standards, by tuning the fractional applied current of each colored primary LED in three-, four-, five-, and six-color multi-package WLEDs. Figure 4 shows the overlapped, integrated emission spectra of the multi-package WLEDs with an increased number of monochromatic LEDs at 2000, 2700, 3500, 4500, 5000, 6500, and 10000 K. The total applied current of the multi-package WLED was varied from 60 to 120 mA with an increase in the number of color LEDs. As previously reported^17, 18^, the fractional current of a long-wavelength colored DC-LED in any WLED gradually increases as the CCT of the white color decreases. The fraction current of a short-wavelength colored DC-LED in multi-package WLEDs increases as the CCT increases. Any specified white or mixed colors in the color gamut area of multi-package WLEDs in the CIE diagram can be realized by dynamically controlling the fractional applied current of each monochromatic LED in the multi-package WLEDs. However, it can be seen that white lights with similar CCT values have different white SPDs among the three-, four-, five-, and six-color multi-package WLEDs. Figure 4 indicates that the color gaps between the monochromatic LED peaks decrease with an increase in the number of PeQD-based monochromatic DC-LEDs in the multi-package WLEDs. Among the four types of multi-package WLEDs, the BCGYOR six-color multi-package WLED can reproduce any color in the wavelength range of the V(λ) and C(λ) curves and the color-matching curves [i.e., the short (S), medium (M), and long (L) types for monochromatic spectral stimuli] of visible light (see Figure S4)^3, 21, 22^. Owing to the narrow bandwidth of PeQDs, at least six multi-package WLEDs can cover the entire visual, circadian and color-sensitivity ranges of the human eye in visible light, indicating that controlling six individual LEDs can reproduce any color of an actual object and the circadian tunable capability under any CCT of multi-package WLEDs.

The insets in Fig. 4 show actual images of white colors from BGR tri-color, BGAR four-color, BCGAR five-color and BCGYOR six-color PeQD-containing multi-package WLEDs with CCTs of 6500 and 3500 K. These images indicate that the four types of white colors have different color tones and hues even at similar CCT levels due to the slightly different SPDs of the white colors. As previously indicated^3^, the different shapes of the SPDs provide different electro-optical performance values (LER, CER, CAF, CRI, CFI, CGI, and the color icon graph) at similar CCT levels. Instead of the currently used CRIs, new color metrics, in this case the CFI score, CGI score, and the color icon graph are used to assess the precise ability of any WLED to reproduce the actual colors of objects compared to ideal light, such as sunlight (CCT ≥ 5500 K), a proportional blend of sunlight, and Planckian locus radiation (4500 < CCT < 5500 K) or Planckian locus radiation ( ≤ 4500 K). Table 1 summarizes the calculated electro-optical performances of the four types of PeQD-containing WLEDs, the BGR tri-color, BGAR four-color, BCGAR five-color and BCGYOR six-color multi-package WLEDs fabricated in this experiment. This table indicates that the LER values of the multi-package WLEDs increase with an increase in the number of monochromatic LEDs. In detail, the LERs increase from 170, 189, 198, 201, 207, 210, and 203 lm/W_opt_ for the tri-color WLED to 270, 277, 287, 285, 289, 277 and 265 lm/W_opt_ for the six-color multi-package WLED at 2000, 2700, 3500, 4500, 5000, 6500, and 10000 K, respectively. Thus, the average LER value for the six-color PeQD-containing WLED increases by ~41% compared with that of the tri-color PeQD-containing WLED. Likewise, the *R*_*a*_ scores show a numerical trend of the multi-package sample similar to that of the LER score, reaching maximum values of 94–96 in the white CCT range between 10000 and 2000 K. The higher values of the LER and *R*_*a*_ scores for the increased number of multi-package WLEDs can be attributed to the fact that the overlapped area of SPDs and the V(λ) curve increase with an increase in the number of packages.

**Table 1:** Optical properties of the three- to six-package white LEDs.

Sample	CCT	CIE x	CIE y	LE (lm/W)	LER (lm/W_opt_)	EQE	CRI (R_a_)	CFI (R_f_)	CGI (R_g_)	CER (blm/W_opt_)	CAF (blm/lm)	CLE (blm/W)
3-PKG (BGR)	2025	0.522	0.411	30.0	170	0.177	1	21.9	118.6	57.6	0.3	10.3
	2825	0.451	0.410	36.6	189	0.194	2	29.1	128.4	89.4	0.5	17.6
	3598	0.400	0.389	41.3	198	0.209	5	33.4	131.7	119.3	0.6	25.3
	4387	0.365	0.370	42.8	201	0.213	7	34.4	132	144.1	0.7	31.2
	5158	0.341	0.359	47.7	207	0.230	10	38.5	131.6	163.9	0.8	38.3
	6500	0.312	0.338	51.9	210	0.247	19	40.6	130.5	191.8	0.9	48.1
	10000	0.278	0.293	54.9	203	0.271	27	41.7	127.7	235.6	1.2	64.9
4-PKG (BGAR)	2012	0.529	0.418	38.9	238	0.163	81	82.2	111	52.3	0.2	8.6
	2682	0.454	0.398	45.1	246	0.183	72	76.2	119.8	99.2	0.4	18.4
	3322	0.416	0.396	50.1	255	0.197	67	74.4	119	125.0	0.5	24.9
	4466	0.361	0.359	55.7	251	0.222	64	71.4	120.2	172.3	0.7	38.7
	5079	0.343	0.352	58.5	252	0.232	63	70.8	120.2	188.7	0.8	44.4
	6292	0.316	0.337	64.2	253	0.254	65	69.9	119	216.9	0.9	55.8
	9727	0.280	0.296	69.4	240	0.289	66	68.6	117.3	263.8	1.1	77.4
5-PKG (BCGAR)	2128	0.510	0.411	40.3	261	0.155	86	86.4	98.3	72.0	0.3	11.2
	2711	0.459	0.410	44.8	262	0.171	88	83.5	96.9	114.6	0.4	19.8
	3258	0.418	0.394	48.1	261	0.184	83	82.6	101.2	146.9	0.6	27.4
	4588	0.359	0.369	55.2	262	0.210	81	80.9	103.6	200.0	0.8	42.6
	5132	0.342	0.356	56.3	260	0.217	81	81.8	105.3	216.7	0.8	47.7
	6500	0.312	0.337	60.0	255	0.236	81	80.1	104.2	251.7	1.0	60.2
	9727	0.278	0.300	64.3	243	0.265	84	81.4	104.6	294.6	1.2	79.2
6-PKG (BCGYOR)	2128	0.512	0.413	38.8	270	0.144	95	87.3	92.3	79.3	0.3	11.5
	2605	0.463	0.403	43.3	277	0.156	96	93.6	100.8	114.4	0.4	18.1
	3312	0.417	0.397	48.1	287	0.168	94	92	101.1	151.6	0.5	25.7
	4387	0.366	0.371	53.2	285	0.187	94	90.3	101.3	201.5	0.7	38.1
	4952	0.348	0.368	55.7	289	0.193	95	91.3	100.9	217.8	0.8	42.5
	6459	0.313	0.334	58.8	277	0.212	95	91.4	102	260.3	1.0	56.0
	10556	0.273	0.294	61.4	265	0.231	96	90.5	98.5	317.0	1.2	74.3

**Figure 5:**
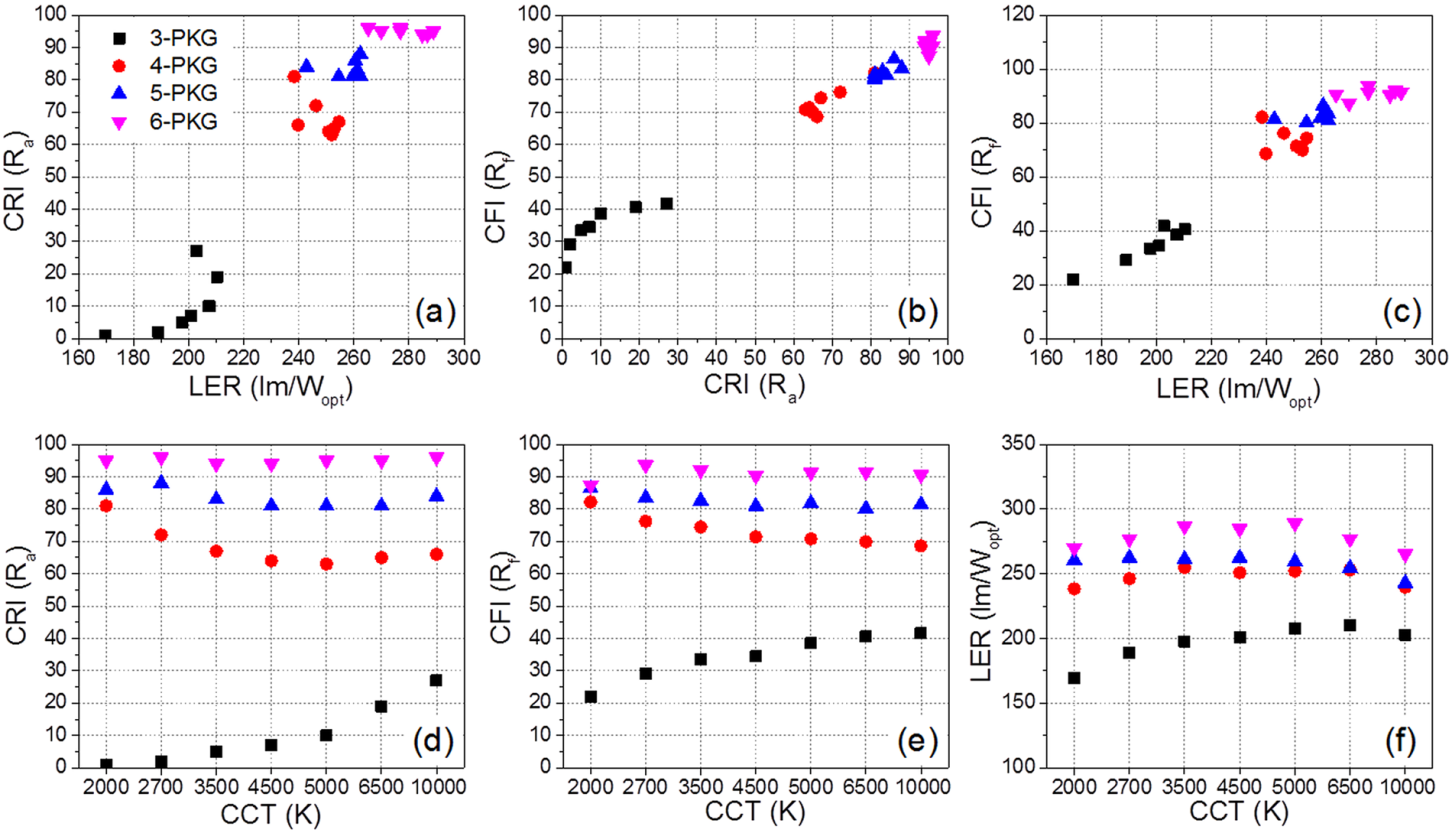
The relationships among (**a**) CRI-LER, (**b**) CFI-CRI, (**c**) CFI-LER, (**d**) CRI-CCT, (**e**) CFI-CCT, and (**f**) LER-CCT of the three- to six-package WLEDs.

As indicated by the relationship between *R*_*a*_ and LER for the PeQD-containing multi-package WLEDs shown in Fig. 5a, an increase in the number of multi-package WLEDs can be a promising solution to increase the LER and CRI scores simultaneously. In addition, this figure clearly indicates that the six-color narrow-band PeQD-containing WLEDs satisfy the requirements of improved visual energy efficiency (LER) and improved color rendition (*R*_*a*_) values simultaneously. However, Table 1 and Fig. 5b indicate that the relationship between the *R*_*f*_ and *R*_*a*_ scores differ from that expected. It was found that the *R*_*a*_ scores of the tri-color and four-color WLEDs are lower than the *R*_*f*_ scores. Both the *R*_*f*_ and the *R*_*a*_ scores of the five-color WLEDs are similar in the white CCT range between 10000 and 2000 K. Otherwise, the *R*_*a*_ scores (94–96) of the six-color WLEDs are higher than the *R*_*f*_ scores (87–94) in the white CCT range between 10000 and 2000 K. It can be considered that the *R*_*a*_ score is overestimated over 90 and underestimated below 70. Consistent with previous reports, the use of the *R*_*f*_ score can be beneficial to determine the optimized SPD of white light from any type of white lighting. This marks the first report showing that the *R*_*f*_ scores of six-color WLEDs exceed 90 in the white CCT range between 10000 and 2700 K, with the exception being the score of 87.3 at 2000 K. Therefore, six-color multi-package WLEDs exhibit the highest ever *R*_*f*_ scores in nearly all of the reported DC-WLEDs and allow individuals to perceive any color in the wavelength range of the V(λ) curve of visible light, namely, close to sunlight. Figure 5c also shows that the relationship between *R*_*f*_ and LER in the multi-package WLED is similar to that between *R*_*a*_ and LER, although the relationship between the *R*_*a*_ and *R*_*f*_ scores deviates from 1:1 linearity.

In an attempt to develop a novel narrow-band PeQD by determining the number of colored QDs and the peak wavelength of each QD, it is considered necessary to increase both the color rendition (*R*_*f*_) and the visual energy efficiency (LER) simultaneously until all six colors can be incorporated into the narrow-band PeQD-based, multi-package WLEDs. Figure 5d,f also show the trends of the *R*_*a*_, *R*_*f*_, and LER scores of the multi-package WLEDs as a function of the CCT of white light. The three- and four-package PeQD WLEDs have large variations in all cases of the *R*_*a*_, *R*_*f*_, and LER scores with a change of the CCT due to the large color gaps between the monochromatic peaks. Otherwise, the five- and six-color multi-package samples have small variations of the *R*_*a*_, *R*_*f*_, and LER scores with a change of CCT due to the decreased color gaps between the monochromatic peaks. The closed color gap of the WLEDs can reduce the variation in the color quality, even with wide variation of the CCTs. All *R*_*a*_, *R*_*f*_, and LER values of the six-color WLEDs are similar and show no distinct trend according to variations in the CCT of the multi-package WLEDs. All data indicate that the six-color WLEDs show the highest values and the fewest variations in the *R*_*a*_, *R*_*f*_, and LER values among the multi-package WLEDs in the three- and six-color package samples.

**Figure 6:**
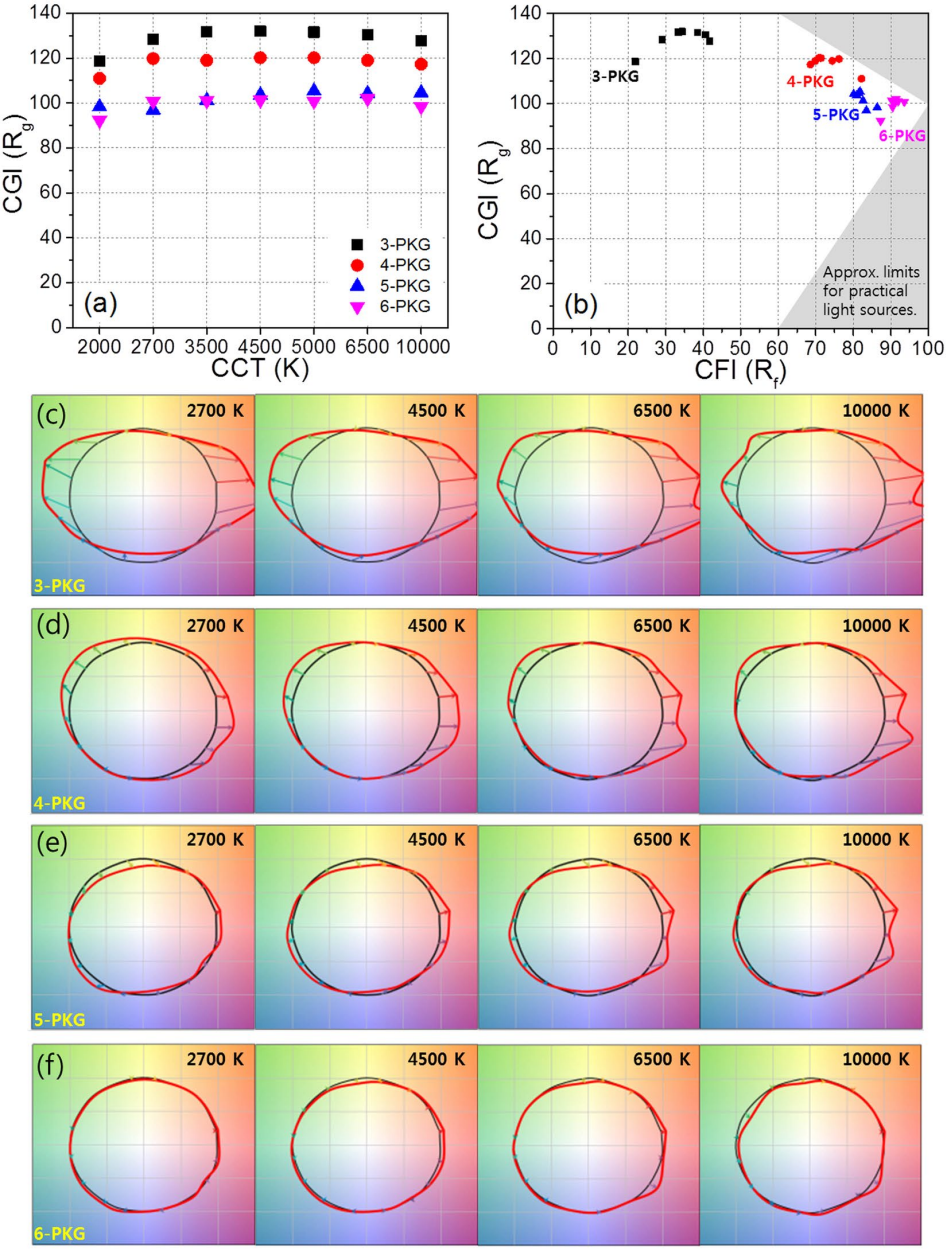
The relationship of (**a**) CGI–CCT, and (**b**) CGI-CFI among the three- to six-package WLEDs. Color icons of the (**c**) three-, (**d**) four-, (**e**) five-, and (**f**) six-package WLEDs at 2700, 4500, 6500, and 10000 K.

As shown in Table 1 and Fig. 6a, the *R*_*g*_ values ranged from 118.6 to 132, indicating that the tri-package WLEDs render an average color that is more saturated than the reference illuminant at any CCT in the white CCT range between 10000 and 2000 K. In addition, *R*_*g*_ values between 98.5 and 102 mean that the six-color WLED renders an average color that is well matched to the reference color of each CCT in the white CCT range between 10000 and 2700 K, but not at 2000 K. As previously reported^7, 8^, color icons (color vector graph, Fig. 6c–f) can be used to explain variation trends systematically for both the hue and the saturation regarding how 16 colors change with an increased number of multi-package WLEDs incorporated with narrow-band colored PeQDs. The bluish-green and violet-red colors and hues of the tri-package WLED are too greatly oversaturated and shifted. Otherwise, the six-package WLEDs indicate that the hue shift and saturation change of the 16 colors show the smallest amount of change with the CCT in the white CCT range between 10000 and 2700 K. These figures also indicate that the color and hue distortion of multi-package WLEDs decrease with an increase in the number of monochromatic packages in multi-package WLEDs.

Figure 6b shows an *R*_*g*_–*R*_*f*_ space plot of multi-package PeQD-containing WLEDs with which to identify which types of PeQD-based LED lightings are feasible at certain color distortions and in certain hue ranges. If the *R*_*g*_–*R*_*f*_ scores are located in the preferred triangular zone within the ranges of *R*_*f*_ > 90 and 90 ≤ *R*_*g*_ ≤ 110, the values in the triangle could provide a guideline for appropriate materials for better lighting types close to the reference illuminant. In this figure, it can be seen that the six-package WLED makes it possible to reach the preferred triangular zone. This figure also indicates that the shape of the scattered *R*_*g*_–*R*_*f*_ scores of the six-package PeQD containing WLED is linear in the white CCT range between 10000 and 2700 K, but not at the case of 2000 K. It can be speculated that the right end of the line, which is displayed as a function of the CCT, indicates the best value of the *R*_*g*_–*R*_*f*_ scores of the six-package WLEDs for typical warm white with a CCT of 2700 K.

**Figure 7:**
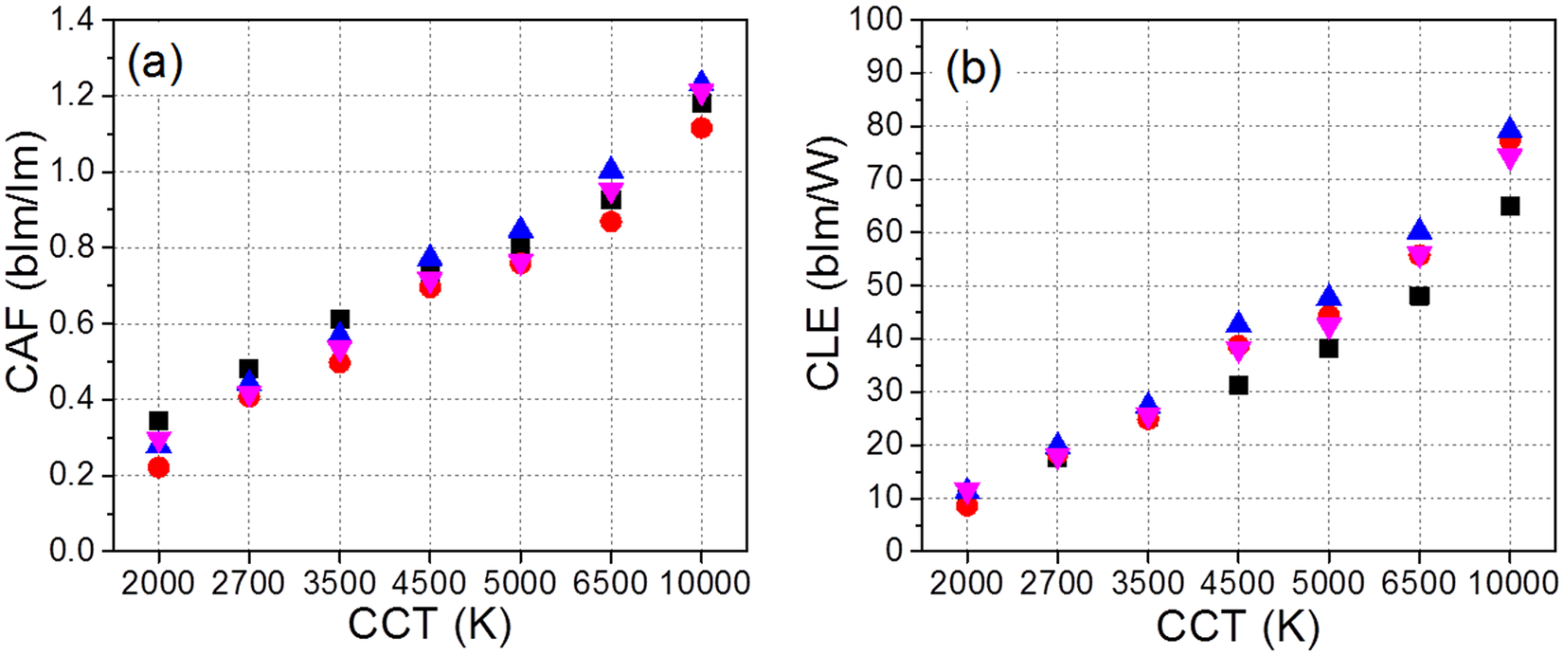
The relationship of (**a**) CAF-CCT, and (**b**) CLE-CCT among the three- to six-package WLEDs.

We also compare the circadian performances, in this case the CAF and CLE values, of multi-package PeQD-containing WLEDs in the white CCT range between 10000 and 2000 K. As expected, both the CAF and the CER values vary from warm white to cool white irrespective of the number of monochromatic LEDs in the multi-package WLED. As shown in Table 1 and Fig. 7, the tunability ranges of the CAF and CLE values indicate that the six-package WLED provides the widest values between warm and cool white lightings. The large circadian differences in the CAF and CER values between white light rich in blue (10000 K) and white light devoid of blue (2000 K) can provide more tunable lighting in terms of the effect on human health. As previously defined^3^, the threshold levels for the activation of the circadian system can be measured on a circadian illuminance (CIL) scale, and the relative melatonin suppression values (MSV) can also be directly related to the CIL values of the lighting. Therefore, wide tunability of CIL values is a very important requirement to control the circadian effect of lighting on human health. In addition, CIL values represent brightness in a circadian sense based on the circadian sensitivity curve, C(λ), of retinal ganglion cells, and similar CIL values have similar circadian effects regardless of the types of light sources. Light with a low CIL value but that maintains high visual illuminance (VIL) is desirable for night lighting, but such light has never been effectively commercialized considering the CIL. A low CIL value and high VIL value can be realized for night light with the low CCT lamp (2000 K) of the multi-package WLEDs, as low CCT lamps have high LERs and low CERs at the lowest CCT value. Accordingly, high LER and low CAF values are required for a good night light candidate as a circadian-controlled lamp to activate melatonin secretion. It can be considered that both a LER value of 270 lm/W_opt_ and a CAF value of 0.3 of the six-package WLED at 2000 K are promising values for night lighting among the four types of multi-package WLEDs in this experiment. Otherwise, a high CIL value and a high VIL value can be realized for daytime light with the high CCT lamp (10000 K) of the multi-package WLEDs. Thus, both high LER and high CAF values are prerequisites for a good candidate daytime light as a circadian-controlled lamp to suppress melatonin secretion. Similarly, both a LER value of 265 lm/W_opt_ and a CAF value of 1.2 for the six-package WLED at 10000 K are the best values for daytime light among the four types of multi-package WLEDs tested in this study.

## Conclusion

New color metrics for evaluating the color rendition of DC-WLEDs, in this case a two-measure system (*R*_*f*_, *R*_*g*_, and color icons), and health metrics for analyzing the circadian figures of merit (CER and CAF) have been suggested to guide the optimization of future lighting types. Here, the newly developed color rendition metrics and circadian metrics are also proposed as new and critical criteria with which to optimize color-converting materials such as QDs as well as inorganic phosphors for warm and cool DC-WLEDs with high visual energy efficiency levels. This approach is appropriate for the creation of new criteria for optimizing the design, number, and peak wavelength of narrow-band QDs for the tuning of the crystal structure, dot size, degree of any alloy used, and the available coating number and thickness of a shelling material.

We successfully synthesized six CGYAOR-emitting CsPb(Br_1−x_, X_x_)_3_ PeQDs at 200 °C as ternary and quaternary CsPb(Br_0.75_, Cl_0.25_)_3_ cyan, CsPbBr_3_ green, CsPb(Br_0.65_, I_0.35_)_3_ yellowish green, CsPb(Br_0.6_, I_0.4_)_3_ amber, CsPb(Br_0.5_, I_0.5_)_3_, orange, and CsPb(Br_0.35_, I_0.65_)_3_ red PeQD color converters. The PL optical properties of the six selected CGYAOR-emitting CsPb(Br_1−x_, X_x_)_3_ PeQDs revealed color emission wavelengths of 488, 517, 553, 567, 585, and 644 nm, PLQYs of 0.64, 0.80, 0.48, 0.50, 0.53, and 0.72, and FWHM values of 16, 17, 22, 24, 27, and 31 nm, respectively. Pure, monochromatic CGYAOR PeQD-containing LEDs were fabricated by combining the five colored PeQDs and a UV-curable polyurethane-based NOA 63 binder capped with a blue-mirror-yellow-window LPDF. The fabricated CGYAOR-emitting monochromatic DC-LEDs provide corresponding LE values of 49, 169, 86, 100, 86, and 25 lm/W at an applied current of 20 mA. Among BGR tricolor, BGAR four-color, BCGAR five-color and BCGYOR six-color WLEDs, the BCGYOR six-color WLED provides the best LERs (270, 277, 287, 285, 289, 277 and 265 lm/W_opt_), CRIs, (95, 96, 94, 94, 95, 95, and 96) and CFIs (87.3, 93.6, 92, 90.3, 91.3, 91.4, and 90.5) along with well-matched CGIs (92.3, 100.8, 101.1, 101.3, 100.9, 102.0, and 98.5) and color icon graphs relative to those of a reference light at a series of CCTs (2000, 2700, 3500, 4500, 5000, 6500, and 10000 K) with a total applied current of 120 mA. In addition, the BCGYOR six-color PeQD-containing WLED provides wide tunable ranges of the circadian performances [CER (79.3–317.0 blm/W_opt_) and CAF (0.3–1.2)] at similar LERs (270–289 lm/W_opt_) with a change of the CCT (2000–10000 K). It is clearly seen that ideal values of the LER, *R*_*g*_, *R*_*f*_, the color icons, the *R*_*g*_–*R*_*f*_ space plot, and the wide tunability of CER and CAF for both daytime and nighttime can be achieved using the BCGYOR six-package PeQD-containing WLED. Accordingly, the new color metrics, visual energy performance capabilities, and the circadian criteria of the six-color WLEDs make it possible to realize a future BCGYOR six-package PeQD-containing WLED as a promising human-centric lighting type for maximizing the visual energy, color qualities, and health effects.

## Methods

### Preparation of colloidal CsPb(Br_1−x_, X_x_)_3_ PeQDs

Colloidal CsPb(Br_1−x_, X_x_)_3_ perovskite QDs can be synthesized by following a previously reported protocol^13^. To synthesize CsPb(Br_1−x_, X_x_)_3_ perovskite QDs, a Cs-oleate solution, involving a mixture of 0.32 g of Cs_2_CO_3_, 12 mL of octadecene (ODE), and 0.1 mL of oleic acid (OA) was prepared at 150 °C with stirring and N_2_ purging until the Cs_2_CO_3_ white powder was completely dissolved in the solution. A total of 5 mL of ODE and 0.188 mmol PbBr_2_ (for green CsPbBr_3_) or a mixed powder Pb Br_2_/PbCl_2_ and PbBr_2_/PbI_2_ [for other colored CsPb(Br_1−x_, X_x_)_3_] with moderate molar ratios were loaded into a three-necked flask. The mixture was degassed under stirring for 1 h at 120 °C and filled with N_2_ gas. Sequentially, 0.5 ml of oleylamine (OLA) and 0.5 ml of OA were injected into the mixture and the solution was sufficiently reacted until Pb-halide precursors were dissolved in the solution (about 10 min). The as-obtained 0.4 ml of Cs-oleate solution was then swiftly injected at 200 °C into the reaction flask. After 5 s, the reaction flask was cooled in an ice-water bath to quench the CsPb(Br_1-x_, X_x_)_3_ solution. The CsPb(Br_1−x_, X_x_)_3_ crude solution was then centrifuged at 12,000 rpm for 5 min and the precipitate was dispersed in 13 mL of hexane. After another session of centrifugation under the same conditions, the cleared CsPb(Br_1−x_, X_x_)_3_ solution was stored in a desiccator for further experiments.

### Fabrication of CsPb(Br_1−x_, X_x_)_3_ PeQD-based monochromatic DC-LEDs

The CsPb(Br_1−x_, X_x_)_3_ solution used here was prepared with an optical density (OD) of 1.5 at 488 nm for CsPb(Br_0.75_, Cl_0.25_)_3_, 511 nm for CsPbBr_3_, 553 nm for CsPb(Br_0.65_, I_0.35_)_3_, 565 nm for CsPb(Br_0.6_, I_0.4_)_3_, 590 nm for CsPb(Br_0.5_, I_0.5_)_3_, and 643 nm for CsPb(Br_0.35_, I_0.65_)_3_ using a UV-Vis spectrometer^17^. A UV-curable binder [Norland Optical Adhesive 63^®^ (NOA 63)] was dissolved in toluene at 4 wt%. Subsequently, 1 mL CsPb(Br_1−x_, X_x_)_3_ and 0.27 mL of NOA 63 solution were mixed in a 5 mL vial. The PeQD/NOA 63 mixture was then placed under a vacuum for 3 h to evaporate the solvents. The residual PeQD/NOA 63 paste was loaded onto a cup-type InGaN blue LED and placed under 365 nm UV-light for 30 min. To obtain the saturated-color monochromatic LED, a long-wavelength pass-dichroic filter (LPDF) was capped onto the DC-LED packages.

### Characterization

The optical properties of CsPb(Br_1−x_, X_x_)_3_ QDs could be obtained by utilizing a UV-Vis spectrometer (S-3100, SINCO Co., Ltd) for absorbance and a spectrophotometer (Darsa, PSI Trading Co., Ltd.) for photoluminescence. The quantum yields (QYs) of the colloidal CsPb(Br_1−x_, X_x_)_3_ QDs were obtained by calculation with a commercial dye (Rhodamine 6 G, QY = 0.95 in ethanol). The optical properties of the CsPb(Br_1−x_, X_x_)_3_ PeQDs-based monochromatic DC-LEDs and multi-package WLEDs were measured by an integrated sphere with a spectrophotometer (Darsapro-5000, PSI Trading Co., Ltd.).

## Electronic supplementary material

SUPPLEMENTARY INFORMATION FOR: Circadian-tunable Perovskite Quantum Dot-based Down-Converted Multi-package White LED with a Color Fidelity Index over 90
