# Supplementary material for: Circadian-tunable Perovskite Quantum Dot-based Down-Converted Multi-Package White LED with a Color Fidelity Index over 90
Source: Sci Rep. 2017 Jun 5;7:2808. doi: 10.1038/s41598-017-03063-7 (PMC5459832; doi:10.1038/s41598-017-03063-7)
Supplement: Supplementary file 1 — SUPPLEMENTARY INFORMATION FOR: Circadian-tunable Perovskite Quantum Dot-based Down-Converted Multi-package White LED with a Color Fidelity Index over 90 [file 41598_2017_3063_MOESM1_ESM.pdf]

SUPPLEMENTARY INFORMATION FOR:

Circadian-tunable Perovskite Quantum Dot-based Down-Converted Multi-package White LED with a Color Fidelity Index over 90

Hee Chang Yoon[1], Ji Hye Oh[1], Soyoung Lee[1], Jae Byung Park[2,*], and Young Rag Do[1,*]

[†]Department of Chemistry, Kookmin University, Seoul 136-702, Republic of Korea
[‡]Department of Display and Semiconductor Physics, Korea University, Sejong, 30019, Republic of Korea

*E-mail: jaebyungpark@korea.ac.kr, yrdo@kookmin.ac.kr

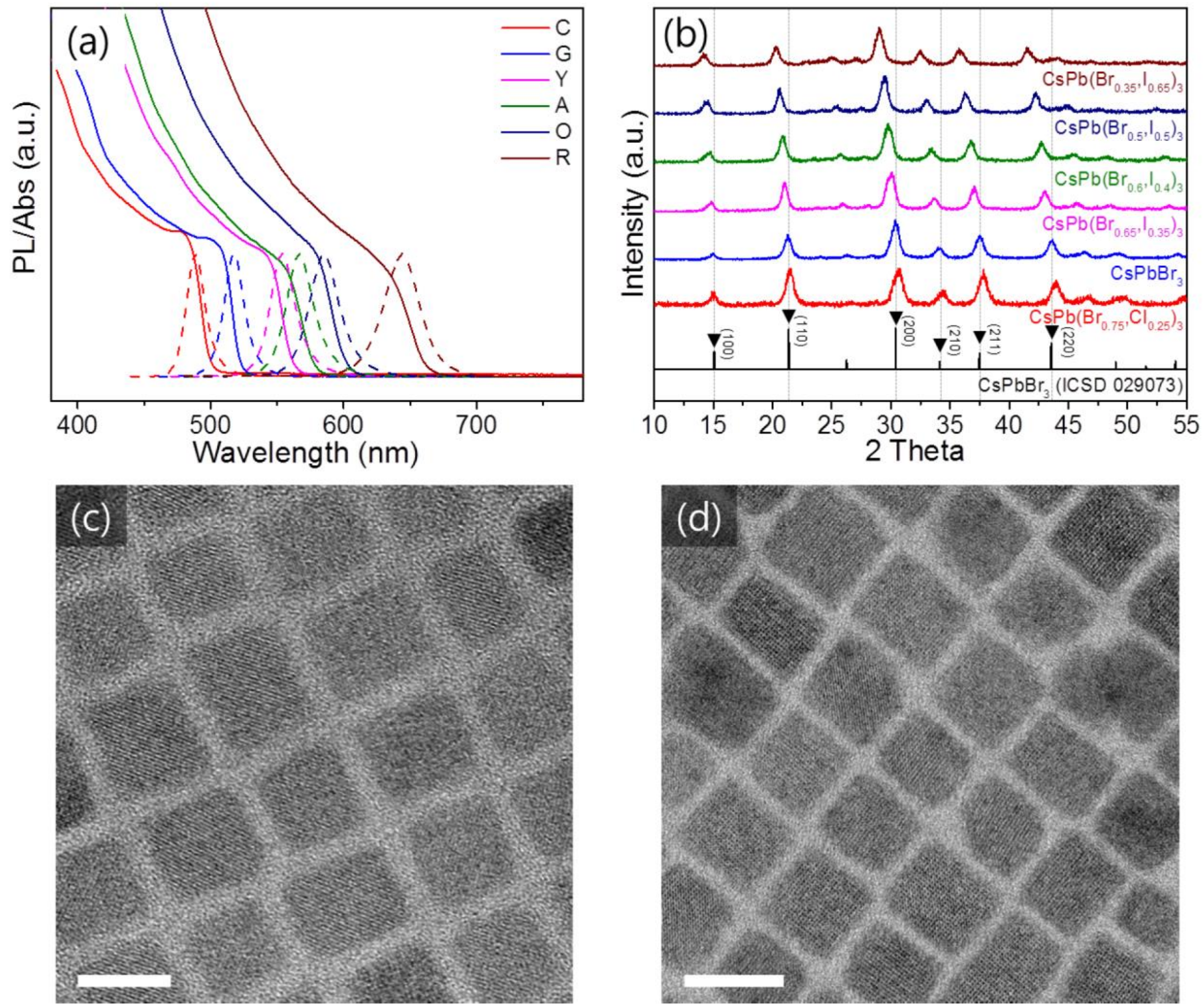

Figure S1. (a) Optical properties (dash line – PL spectra, solid line - absorbance), (b) XRD peak of CGYAOR color $CsPb(Br_{1-x}I_x)_3$ PeQDs, TEM images of (c) green emissive $CsPbBr_3$ PeQD and (d) red emissive $CsPb(Br_{0.35}I_{0.65})_3$ PeQD with 10 nm white bar

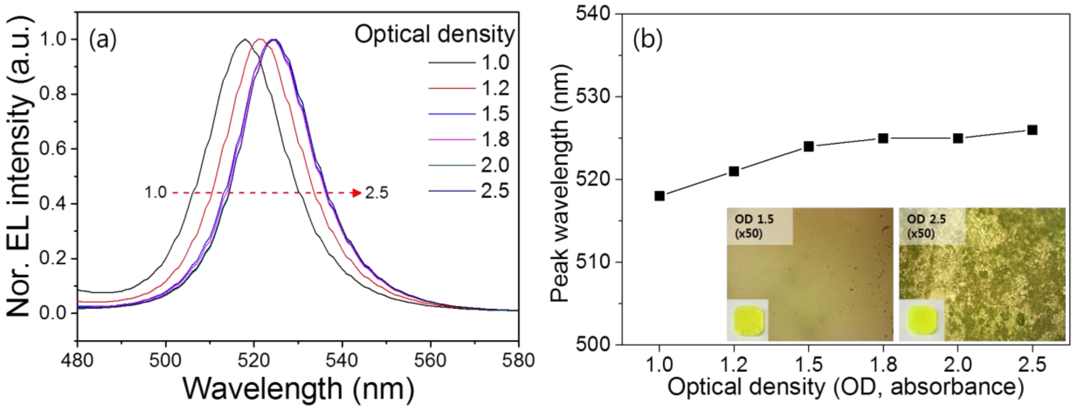

Figure S2. (a) The normalized EL spectra, and (b) the peak wavelength (inset: optical microscope images, x50 of magnification) of $CsPbBr_3$ PeQD/NOA packaged DC-LEDs as a function of the optical density of the PeQDs ranging from 1.0 to 2.5 at 511 nm. The $CsPbBr_3$ PeQDs DC-LEDs were measured without a LPDF in an integrated sphere with a spectrophotometer. With an increase in the PeQD concentration (increasing the optical density of the PeQDs), the emission wavelength of the $CsPbBr_3$ PeQDs DC-LEDs became longer (red-shift) due to the agglomeration of the PeQDs and the energy transfers among neighboring PeQDs. The inset of Figure S2(b) shows the agglomeration of PeQDs under the high-OD condition.

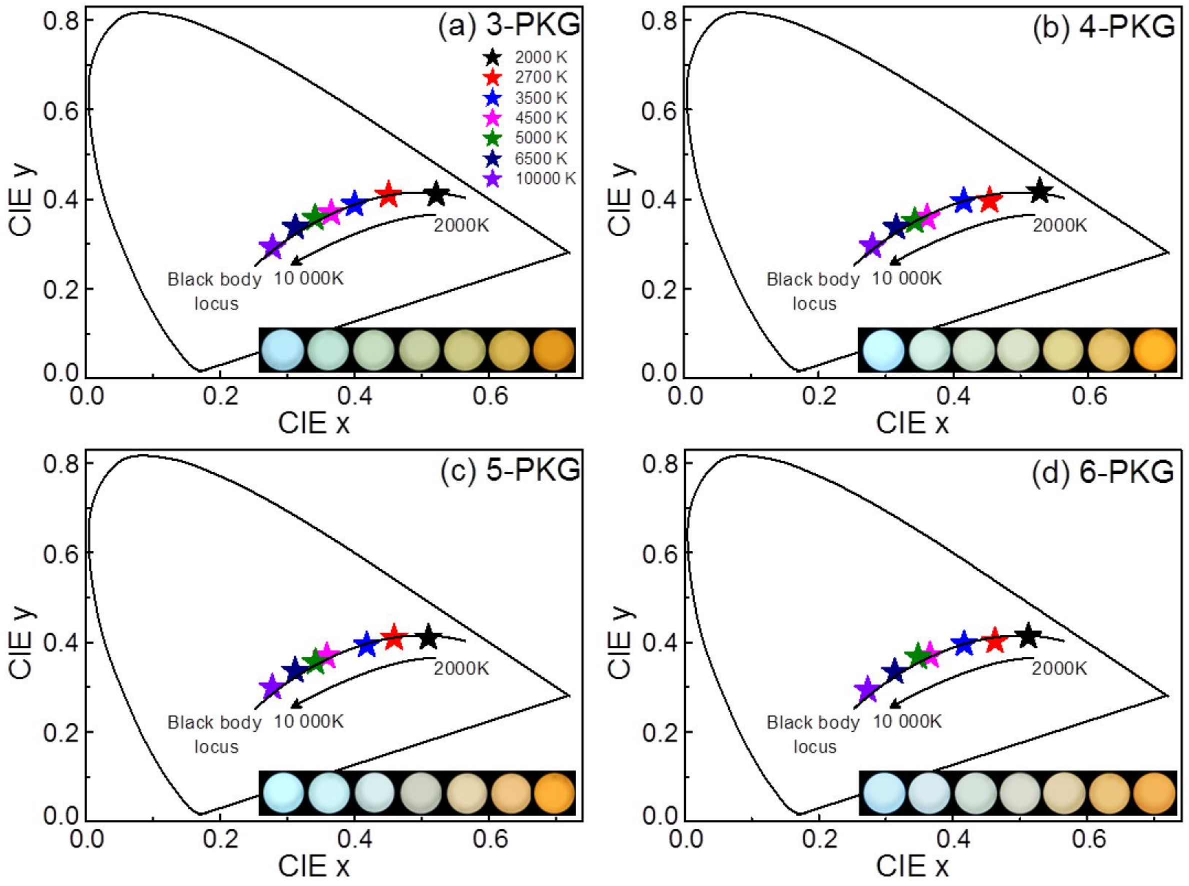

Figure S3. CIE color coordinates of (a) three-, (b) four-, (c) five-, and (d) six-packaged white LED as white CCT range between 10,000 K and 2000 K. Insets show emission photographs of each multi-package white light from black-body sphere with increasing the CCT from right to left.

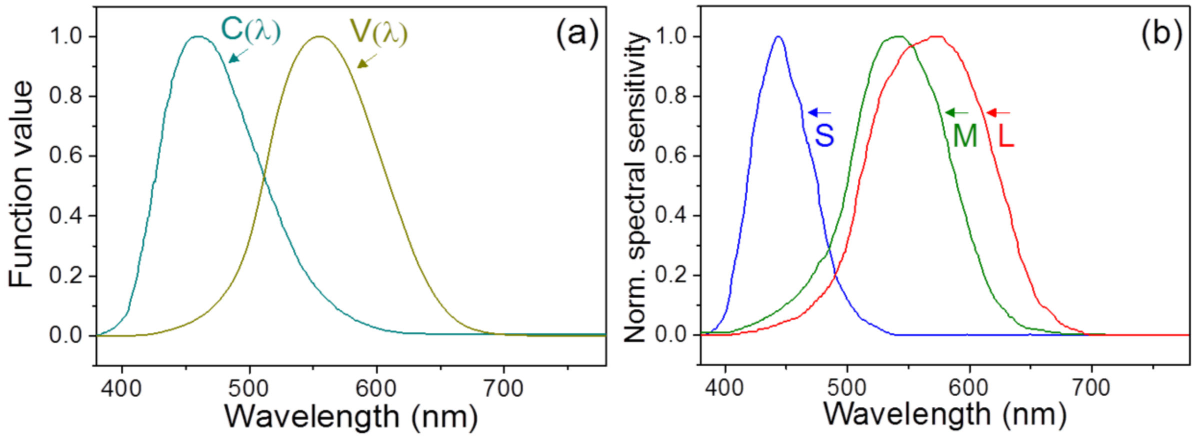

Figure S4. (a) The V(λ) and C(λ) curves, and (b) the color matching curves of monochromatic spectral stimuli in the short (S), medium (M), and long (L) type cone cell.

Table S1. Optical properties of $CsPb(Br_{0.75},Cl_{0.25})_3$ cyan, $CsPbBr_3$ green, $CsPb(Br_{0.65},I_{0.35})_3$ yellowish green, $CsPb(Br_{0.6},I_{0.4})_3$ amber, $CsPb(Br_{0.5},I_{0.5})_3$ orange, and $CsPb(Br_{0.35},I_{0.65})_3$ red PeQD containing monochromatic DC-LED and InGaN blue-chip LED at an applied current of 20 mA.

Sample	CIE x	CIE y	LE (lm/W)	LER (lm/W)	EQE	Peak wavelength (nm)	FWHM (nm)
Blue LED	0.152	0.026	17	35	0.486	450	21
$CsPb(Br_{0.75}Cl_{0.25})_3$ C	0.090	0.295	49	188	0.258	489	21
$CsPbBr_3$ G	0.153	0.742	169	527	0.321	525	19
$CsPb(Br_{0.65}I_{0.35})_3$ Y	0.430	0.564	86	613	0.140	561	23
$CsPb(Br_{0.6}I_{0.4})_3$ A	0.506	0.493	100	569	0.175	574	24
$CsPb(Br_{0.5}I_{0.5})_3$ O	0.580	0.416	86	450	0.191	593	30
$CsPb(Br_{0.35}I_{0.65})_3$ R	0.714	0.286	25	93	0.273	645	28

Table S2. Summary of abbreviations in main text

**Full-name**	**Abbreviation**
Perovskite	Pe
Quantum dots	QDs
Down-converted White-light-emitting-diodes	DC-WLED
Spectral power distribution	SPD
Optical density	OD
Luminous efficacy	LE
Circadian luminous efficacy	CLE
Luminous efficacy of radiation	LER
Circadian efficacy of radiation	CER
Circadian illuminance	CIL
Color rendering index	CRI, R_a
Color fidelity index	CFI, R_f
Color gamut index	CGI, R_g
Circadian action factor	CAF
photoluminescence	PL
Electroluminescence (Electroluminescent)	EL
Quantum yield	QY
Correlated color temperature	CCT
Melatonin suppression value	MSV
Long-wavelength pass-dichroic filter	LPDF
Blue	B
Cyan	C
Green	G
Yellowish green	Y
Amber	A
Orange	O
Red	R
Package	PKG
Full-width at half-maximum	FWHM
Octadecene	ODE
Oleylamine	OLA
Oleic acid	OA
Norland optical adhesive 63®	NOA 63
